# Association of blood urea nitrogen to creatinine ratio with incident type 2 diabetes mellitus: A retrospective cohort study in the Chinese population

**DOI:** 10.1097/MD.0000000000039003

**Published:** 2024-07-26

**Authors:** Xiuping Yin, Yiguo Wang, Jianjun Jiang, Fengxing Zhong, Qiming Zhang

**Affiliations:** a Experimental Research Center, China Academy of Chinese Medical Sciences, Beijing, China; b Department of Traditional Chinese Medicine, ZhongCe Town Health Center, Jining, Shandong Province, China.

**Keywords:** blood urea nitrogen to creatinine ratio, insulin resistance, renal dysfunction, type 2 diabetes mellitus

## Abstract

Renal dysfunction can lead to insulin resistance and increase the incidence of type 2 diabetes mellitus (T2DM). The blood urea nitrogen to creatinine ratio (NCR) is a frequently used indicator to assess renal dysfunction and differentiate between prerenal and intrinsic renal injury. However, the association between NCR and T2DM in the Chinese population remains unclear. Hence, this study aimed to investigate the association between NCR and the incidence of T2DM in the Chinese population. The relationship between NCR and T2DM was examined using the Cox proportional hazards model and curve fitting techniques. In addition, a comprehensive set of sensitivity and subgroup analyses were performed. All results were presented as hazard ratios (HRs) and 95% confidence intervals (CIs). Between 2010 and 2016, 189,416 Chinese people were recruited from the Rich Healthcare Group for this retrospective cohort study. Of the participants, 3755 (19.8%) were diagnosed with T2DM during the follow-up period. After full adjustment, the Cox proportional hazards model revealed a positive connection between NCR and the incidence of T2DM (HR = 1.03, 95% CI: 1.02–1.04, *P* < .001). Compared with individuals with lower NCR Q1 (≤13.536), the multivariate HR for NCR and T2DM in Q2 (13.536–16.256), Q3 (16.256–19.638), Q4 (>19.638) were 1.08 (0.98–1.19), 1.16 (1.05–1.28), 1.39 (1.26–1.53). The higher NCR groups (≥20) had a higher ratio of T2DM (HR = 1.28, 95% CI: 1.18–1.38, *P* < .001) than the lowest NCR group (<20). These findings were validated using sensitivity and subgroup analyses. In conclusion, this study found a positive and independent association between NCR and the incidence of T2DM after adjusting for confounding variables.

## 1. Introduction

Diabetes has become a global issue that affects public health across multiple geographical areas.^[1]^ In 2019, approximately 9.3% of the global population was diagnosed with diabetes. By 2030, this number will increase to 10.2% (578 million), and by 2045, it will increase to 10.9% (700 million).^[2]^ Most of these cases (>90%) were classified as type 2 diabetes mellitus (T2DM).^[2]^ In 2021, China had more adults with T2DM than any other country, with a prevalence of 10.6%.^[3]^ The complications of T2DM can affect many organs and systems of the body, as well as the individual’s quality of life.^[4,5]^ Therefore, early identification of the risk factors for T2DM is crucial to prevent its onset.

The kidneys are key organs in the maintenance of diabetic homeostasis.^[6–9]^ Damage to renal function may lead to insulin resistance and T2DM.^[10–12]^ Researchers have found that chronic kidney disease (CKD) and T2DM have similar risk factors, and CKD can increase the risk of diabetes.^[13]^ Blood urea nitrogen (BUN), serum creatinine (Scr), and the blood urea nitrogen to creatinine ratio (NCR) are universal markers of renal dysfunction.^[13,14]^ Nevertheless, BUN cannot be considered a definitive indicator of renal insufficiency and may be influenced by many factors such as neurohormonal activation, protein intake, and catabolic processes.^[14,15]^ Similarly, extrarenal variables such as sex, age, nutrition, and ethnicity affect Scr levels.^[14]^ Hence, there may be limitations in making predictions based solely on BUN or Scr levels. The NCR is a valuable parameter for mitigating the factors affecting accuracy.^[16]^ It is considered more reliable and precise than measuring Scr or BUN separately. Furthermore, in clinical practice, the NCR has been used to differentiate between prerenal and intrinsic renal injury.^[16,17]^ When NCR ≥ 20 is commonly associated with prerenal diseases, which are conditions that reduce blood perfusion to the kidneys and lead to renal dysfunction.^[18,19]^

Recently, several studies have examined the association between BUN and Scr levels and T2DM.^[20–22]^ A thorough study of NCR’s ability to predict T2DM onset in the Chinese population is yet to be conducted. Hence, this study aimed to explain the relationship between NCR and the incidence of T2DM among the Chinese population.

## 2. Materials and methods

### 2.1. Data source

This study used data from the Dryad Digital Repository (https://doi.org/10.5061/dryad.ft8750v) uploaded by Chen et al.^[23]^ By the terms of services provided by Dryad, the investigators were allowed to utilize the data for secondary analysis. The initial study was approved by the Rich Healthcare Group Review Board. Because this study was retrospective, the requirement for informed consent was waived by the Rich Healthcare Group Review Board. We strictly adhered to the Declaration of Helsinki and Strengthening the Reporting of Observational Studies in Epidemiology (STROBE) guidelines throughout this study.^[24]^

### 2.2. Study population

**Figure 1.**
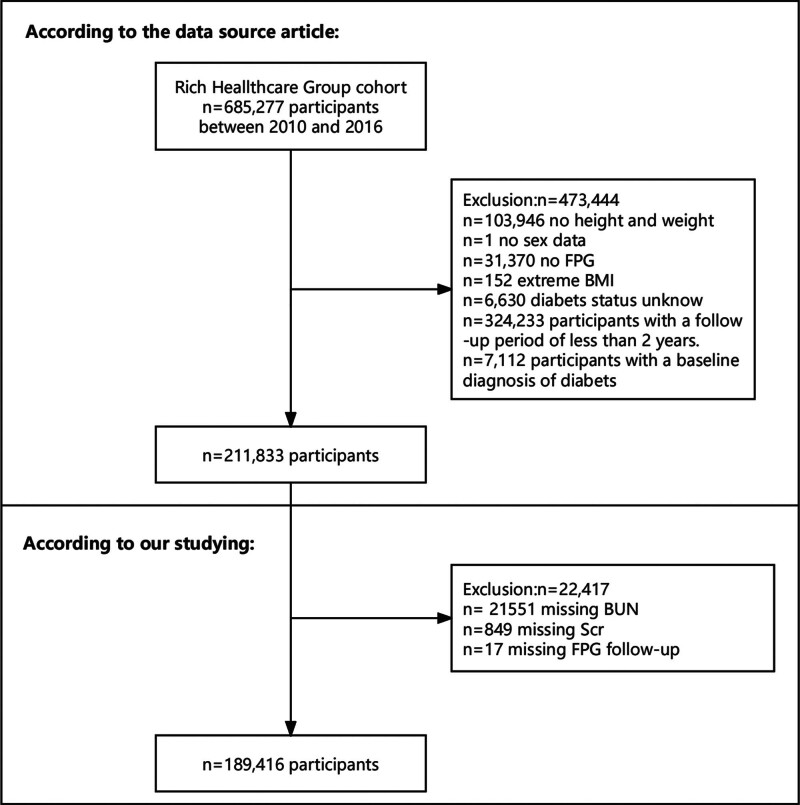
Study population. BMI = body mass index, BUN = blood urea nitrogen, FPG = fasting plasma glucose, Scr = serum creatinine.

The database included 211,833 participants. We excluded participants with missing BUN (n = 21,551), Scr (n = 849), and fasting plasma glucose (FPG; n = 17) data during follow-up. In the secondary analysis, 189,416 individuals were included. A description of the research design and process is shown in Figure 1.

### 2.3. Data collection

Individuals were requested to complete a standardized survey that included critical demographic information such as age and sex, personal health background, living habits (smoking and drinking status), and family history of diabetes. The medical worker used an automated scale to assess the weight and height of the participants and calculated their body mass index (BMI). Participants’ drinking and smoking status were classified into 3 categories: current, ever, and never. Systolic blood pressure (SBP) and diastolic blood pressure (DBP) were assessed using a mercury sphygmomanometer. In addition, a skilled team utilized a Beckman 5800 autoanalyzer (Brea) to gauge various clinical data, including serum triglyceride (TG), low-density lipoprotein cholesterol (LDL-C), FPG, total cholesterol (TC), high-density lipoprotein cholesterol (HDL-C), BUN, Scr, alanine aminotransferase (ALT), and aspartate aminotransferase (AST). The medical staff collected blood samples from all participants who had fasted for at least 10 hours before each examination. The primary continuous variable under investigation was the baseline NCR. The dependent variable was the incidence of T2DM.

### 2.4. Definition

The incidence of T2DM was determined using criteria established by the American Diabetes Association. Participants were classified as patients with T2DM if they had FPG ≥ 7.00 mmol/L or self-reported T2DM diagnosis during the follow-up period.^[23,25,26]^ Participants’ data were reviewed by the medical staff either on the day of T2DM diagnosis or during their last visit.

### 2.5. Statistical analysis

The baseline characteristics of the participants were categorized based on the NCR quartile and T2DM status. Continuous variables were presented as means and standard deviations or medians and interquartile ranges. Categorical variables were expressed as rates and percentages. Continuous variables were compared using a 1-way analysis of variance or Kruskal–Wallis test, and the chi-square test was used to compare categorical data.

For missing values, we used multiple imputations with 5 replicates for the substitution. The number of the missing values of TG, TC, SBP, DBP, AST, ALT, HDL-C, LDL-C, drinking status, and smoking status were 1758 (0.9281%), 1753 (0.9255%), 21 (0.0111%), 22 (0.0116%), 108,456 (57.2581%), 1110 (0.586%), 74,884 (39.5341%), 74,194 (39.1699%), 135,246 (71.4016%), 135,246 (71.4016%), and 124,705 (71.96%), respectively.

We conducted a univariate Cox regression analysis to assess the impact of each variable on the incidence of T2DM. The NCR was classified as low (<20) and high (≥20) based on clinical practice.^[18,27]^ The association between NCR and T2DM was calculated using the Cox proportional hazards model. We developed 4 models: model I, without considering any variables; model II, adjusted only for sex and age; model III, model II + BMI, DBP, SBP, TG, ALT, TC, and family history of diabetes; and model IV, model III + AST, smoking status, and drinking status. All results were presented as hazard ratios (HRs) and 95% confidence intervals (CIs). In addition, we accurately captured the dose–response connection between NCR and T2DM based on model IV using the restricted cubic spline method.

A thorough range of sensitivity analyses were carried out to enhance the reliability of the findings. Studies have indicated an important association between glucose metabolism and specific factors (family history of diabetes, alcohol consumption, and smoking status).^[28,29]^ Therefore, these patients were excluded from multivariate analysis. Approximately 70% of the patients were unable to provide complete smoking and drinking status data, and 50% were unable to provide complete AST data. Therefore, these patients were excluded from multivariate analysis.

We used a stratified Cox proportional hazards model to ensure the consistency of the results. We initially transformed the continuous variables into categorical variables. Subsequently, an interaction test was performed.

Statistical analyses were performed using R 4.4.1 (http://www.R-project.org, The R Foundation, Shanghai, China) and Free Statistics software (Beijing Free Clinical Medical Technology Co. Ltd, Beijing, China), version 1.8.^[30]^

## 3. Results

### 3.1. Baseline characteristics of participants

The final data analysis included 189,416 participants (Fig. 1). The mean age of participants was 42.2 ± 12.7 years, with approximately 55.0% being male. The mean follow-up was 3.1 ± 0.9 years, during which 3755 (19.8%) participants developed T2DM. Baseline characteristics were presented in Tables 1 and 2. Individuals in the NCR of Q4 exhibited higher age, TC, and BUN levels than those in the other 3 groups; this group also had a higher percentage of females and those with a family history of diabetes. Participants in the NCR of Q1 had the highest BMI, DBP, SBP, TG, Scr, ALT, AST, and smoking status (*P* < .001; Table 1). These individuals were divided into 2 groups according to their T2DM status at the most recent follow-up visit. Participants who developed T2DM exhibited characteristics such as older age; a higher proportion of males; and higher SBP, DBP, BMI, TC, TG, LDL-C, BUN, Scr, NCR, ALT, and AST values. Moreover, the percentages of smokers and drinkers were higher in this group (*P* < .001; Table 2).

**Table 1:** Baseline characteristics of study participants.

Variables	Total	Quartile 1 (≤13.536)	Quartile 2 (13.536–16.256)	Quartile 3 (16.256–19.638)	Quartile 4 (>19.638)	*P*-value
Participants	189,416	47,347	47,360	47,355	47,354	
Age (yr)	42.2 ± 12.7	40.2 ± 11.9	41.4 ± 12.3	42.7 ± 12.8	44.5 ± 13.3	<0.001
Sex, n (%)						<0.001
Male	104,245 (55.0)	35,870 (75.8)	29,732 (62.8)	23,911 (50.5)	14,732 (31.1)	
Female	85,171 (45.0)	11,477 (24.2)	17,628 (37.2)	23,444 (49.5)	32,622 (68.9)	
BMI (kg/m^2^)	23.2 ± 3.3	23.5 ± 3.3	23.3 ± 3.3	23.1 ± 3.4	23.0 ± 3.3	<0.001
SBP (mm Hg)	119.1 ± 16.4	120.0 ± 15.7	119.2 ± 16.2	118.8 ± 16.6	118.4 ± 17.1	<0.001
DBP (mm Hg)	74.2 ± 10.8	75.0 ± 10.7	74.4 ± 10.9	74.0 ± 10.9	73.4 ± 10.8	<0.001
TC (mmol/L)	4.7 ± 0.9	4.6 ± 0.9	4.7 ± 0.9	4.7 ± 0.9	4.8 ± 0.9	<0.001
TG (mmol/L)	1.3 ± 1.0	1.5 ± 1.0	1.4 ± 1.0	1.3 ± 1.0	1.2 ± 1.1	<0.001
HDL-C (mmol/L)	1.4 ± 0.3	1.3 ± 0.3	1.3 ± 0.3	1.4 ± 0.3	1.4 ± 0.3	<0.001
LDL-C (mmol/L)	2.8 ± 0.7	2.7 ± 0.7	2.8 ± 0.7	2.8 ± 0.7	2.8 ± 0.7	<0.001
BUN (mmol/L)	4.7 ± 1.2	3.7 ± 0.8	4.4 ± 0.9	4.9 ± 1.0	5.7 ± 1.1	<0.001
Scr (μmol/L)	70.0 ± 15.7	79.5 ± 15.9	72.8 ± 14.4	67.7 ± 13.5	60.1 ± 11.9	<0.001
NCR	17.0 ± 4.8	11.7 ± 1.4	14.9 ± 0.8	17.8 ± 1.0	23.6 ± 3.7	<0.001
ALT (U/L)	18.0 (13.0, 27.6)	20.0 (13.8, 30.1)	18.8 (13.0, 28.9)	17.7 (12.6, 26.9)	16.7 (12.1, 24.5)	<0.001
AST (U/L)	22.0 (18.6, 26.7)	22.3 (19.0, 27.0)	22.0 (18.8, 27.0)	21.8 (18.2, 26.0)	21.4 (18.0, 26.0)	<0.001
Smoking status, n (%)						<0.001
Current smoker	10,863 (20.1)	3605 (23.6)	3097 (21.9)	2517 (19.2)	1644 (14.2)	
Ever smoker	2343 (4.3)	793 (5.2)	704 (5)	556 (4.2)	290 (2.5)	
Never smoker	40,964 (75.6)	10,890 (71.2)	10,342 (73.1)	10,055 (76.6)	9677 (83.3)	
Drinking status, n (%)						<0.001
Current drinker	1214 (2.2)	347 (2.3)	319 (2.3)	325 (2.5)	223 (1.9)	
Ever drinker	8278 (15.3)	2767 (18.1)	2394 (16.9)	1926 (14.7)	1191 (10.3)	
Never drinker	44,678 (82.5)	12,174 (79.6)	11,430 (80.8)	10,877 (82.9)	10,197 (87.8)	
Family history of diabetes, n (%)	3963 (2.1)	831 (1.8)	952 (2)	1039 (2.2)	1141 (2.4)	<0.001
Follow-up (yr)	3.1 ± 0.9	3.1 ± 0.9	3.1 ± 0.9	3.1 ± 0.9	3.1 ± 1.0	<0.001

ALT = alanine aminotransferase, AST = aspartate aminotransferase, BMI = body mass index, BUN = blood urea nitrogen, DBP = diastolic blood pressure, HDL-C = high-density lipoprotein cholesterol, LDL-C = low-density lipoprotein cholesterol, NCR = blood urea nitrogen to creatinine ratio, SBP = systolic blood pressure, Scr = serum creatinine, TC = total cholesterol, TG = triglyceride.

**Table 2:** Baseline characteristics of subjects with/without T2DM.

Variables	Total (N = 189,416)	Non-T2DM (n = 185661)	T2DM (n = 3755)	*P*-value
Age (yr)	42.2 ± 12.7	41.9 ± 12.6	54.9 ± 13.1	<0.001
Sex, n (%)				<0.001
Male	104,245 (55.0)	101,535 (54.7)	2710 (72.2)	
Female	85,171 (45.0)	84,126 (45.3)	1045 (27.8)	
BMI (kg/m^2^)	23.2 ± 3.3	23.2 ± 3.3	26.2 ± 3.5	<0.001
SBP (mm Hg)	119.1 ± 16.4	118.9 ± 16.3	131.8 ± 18.8	<0.001
DBP (mm Hg)	74.2 ± 10.8	74.1 ± 10.8	80.8 ± 11.9	<0.001
TC (mmol/L)	4.7 ± 0.9	4.7 ± 0.9	5.0 ± 0.9	<0.001
TG (mmol/L)	1.3 ± 1.0	1.3 ± 1.0	2.1 ± 1.5	<0.001
HDL-C (mmol/L)	1.4 ± 0.3	1.4 ± 0.3	1.3 ± 0.3	<0.001
LDL-C (mmol/L)	2.8 ± 0.7	2.8 ± 0.7	2.9 ± 0.7	<0.001
BUN (mmol/L)	4.7 ± 1.2	4.7 ± 1.2	5.0 ± 1.3	<0.001
Scr (μmol/L)	70.0 ± 15.7	70.0 ± 15.7	72.5 ± 15.7	<0.001
NCR	17.0 ± 4.8	17.0 ± 4.8	17.6 ± 5.1	<0.001
ALT (U/L)	18.0 (13.0, 27.6)	18.0 (12.9, 27.2)	26.0 (18.0, 41.0)	<0.001
AST (U/L)	22.0 (18.6, 26.7)	22.0 (18.5, 26.5)	25.0 (21.0, 32.0)	<0.001
Smoking status, n (%)				<0.001
Current smoker	10,863 (20.1)	10,491 (19.7)	372 (35.8)	
Ever smoker	2343 (4.3)	2268 (4.3)	75 (7.2)	
Never smoker	40,964 (75.6)	40,373 (76)	591 (56.9)	
Drinking status, n (%)				<0.001
Current drinker	1214 (2.2)	1170 (2.2)	44 (4.2)	
Ever drinker	8278 (15.3)	8106 (15.3)	172 (16.6)	
Never drinker	44,678 (82.5)	43,856 (82.5)	822 (79.2)	
Family history of diabetes	3963 (2.1)	3804 (2)	159 (4.2)	<0.001
Follow-up (yr)	3.1 ± 0.9	3.1 ± 0.9	3.4 ± 1.0	<0.001

ALT = alanine aminotransferase, AST = aspartate aminotransferase, BMI = = body mass index, BUN = blood urea nitrogen, DBP = diastolic blood pressure, HDL-C = high-density lipoprotein cholesterol, LDL-C = low-density lipoprotein cholesterol, NCR = blood urea nitrogen to creatinine, ratio, SBP = systolic blood pressure, Scr = serum creatinine, TC = total cholesterol, T2DM = type 2 diabetes mellitus, TG = triglyceride.

### 3.2. Univariate analysis

According to the univariate analyses, a positive association was observed between the incidence of T2DM and factors such as NCR, age, DBP, BMI, SBP, TG, AST, TC, ALT, family history of diabetes, smoking, and drinking. In addition, the incidence of T2DM was higher in males than females (Table 3).

**Table 3:** Results of univariate analysis.

Variables	HR (95% CI)	*P*-value
NCR	1.02 (1.02–1.03)	<0.001
Age (yr)	1.07 (1.06–1.07)	<0.001
Sex		
Male	Ref	
Female	0.49 (0.45–0.52)	<0.001
BMI (kg/m^2^)	1.24 (1.23–1.24)	<0.001
SBP (mm Hg)	1.04 (1.04–1.04)	<0.001
DBP (mm Hg)	1.05 (1.04–1.05)	<0.001
TC (mmol/L)	1.42 (1.38–1.47)	<0.001
TG (mmol/L)	1.26 (1.25–1.27)	<0.001
ALT (U/L)	1.00 (1.00–1.00)	<0.001
AST (U/L)	1.01 (1.01–1.01)	0.00053
Smoking status		
Current smoker	Ref	
Ever smoker	0.91 (0.78–1.07)	0.2519
Never smoker	0.43 (0.39–0.46)	<0.001
Drinking status		
Current drinker	Ref	
Ever drinker	0.46 (0.37–0.59)	<0.001
Never drinker	0.46 (0.37–0.54)	<0.001
Family history of diabetes		
No	Ref	
Yes	1.76 (1.50–2.06)	<0.001

ALT = alanine aminotransferase, AST = aspartate aminotransferase, BMI = body mass index, CI = confidence interval, DBP = diastolic blood pressure, HR = hazard ratios, NCR = blood urea nitrogen to creatinine ratio, Ref = reference, SBP = systolic blood pressure, TC = total cholesterol, TG = triglyceride.

### 3.3. The relationship between NCR and T2DM

As shown in Table 4. In the unadjusted model (model I), when NCR was considered as a continuous variable, there was a 2% increase in the incidence of T2DM for every 1-U increase in NCR (HR = 1.02, 95% CI: 1.02–1.03, *P* < .001). This correlation remained consistent in the minimum-adjusted model (model II). Furthermore, in the fully-adjusted model (model IV), every 1-U increase in NCR was connected with a 3% higher incidence of T2DM (HR = 1.03, 95% CI: 1.02–1.04, *P* < .001). Next, we categorized NCR into quartiles to further analyze its relationship with the incidence of T2DM. Compared with individuals with lower NCR Q1 (≤13.536), the multivariate HR for NCR and T2DM in Q2 (13.536–16.256), Q3 (16.256–19.638), Q4 (>19.638) were 1.08 (0.98–1.19), 1.16 (1.05–1.28), 1.39 (1.26–1.53) after adjusting for model IV. In addition, higher NCR groups (≥20) exhibited a significantly higher incidence of T2DM (HR = 1.28, 95% CI: 1.18–1.38, *P* < .001) compared with the lowest NCR group (<20) after adjusting for model IV.

**Table 4:** Correlation between NCR and type 2 diabetes mellitus in diverse models.

Variables	Model I [HR (95% CI]	*P*-value	Model II [HR (95% CI]	*P*-value	Model III [HR (95% CI]	*P*-value	Model IV [HR (95% CI]	*P*-value
NCR	1.02 (1.02–1.03)	<0.001	1.02 (1.02–1.03)	<0.001	1.03 (1.02–1.04)	<0.001	1.03 (1.02–1.04)	<0.001
NCR ≥ 20								
No	Ref		Ref		Ref		Ref	
Yes	1.23 (1.15–1.32)	<0.001	1.29 (1.2–1.39)	<0.001	1.27 (1.18–1.37)	<0.001	1.28 (1.18–1.38)	<0.001
NCR (quartile)								
Q1	Ref		Ref		Ref		Ref	
Q2	1.11 (1–1.22)	0.04	1.09 (0.99–1.2)	0.07	1.09 (0.99–1.2)	0.08	1.08 (0.98–1.19)	0.113
Q3	1.16 (1.05–1.27)	0.003	1.16 (1.05–1.27)	0.003	1.16 (1.06–1.28)	0.002	1.16 (1.05–1.28)	0.002
Q4	1.34 (1.23–1.47)	<0.001	1.42 (1.29–1.56)	<0.001	1.4 (1.27–1.54)	<0.001	1.39 (1.26–1.53)	<0.001
*P*_trend_		<0.001		<0.001		<0.001		<0.001

Model I adjusted for none.

Model II adjusted for age and sex.

Model III adjusted for model II + BMI, SBP, TG, DBP, TC, ALT, family history of diabetes.

Model IV adjusted for model III + AST, smoking status, alcohol consumption.

ALT = alanine aminotransferase, AST = aspartate aminotransferase, BMI = body mass index, CI = confidence interval, DBP = diastolic blood pressure, HR = hazard ratio, NCR = blood urea nitrogen to creatinine ratio, Ref = reference, SBP = systolic blood pressure, TC = total cholesterol, TG = triglyceride.

**Figure 2.**
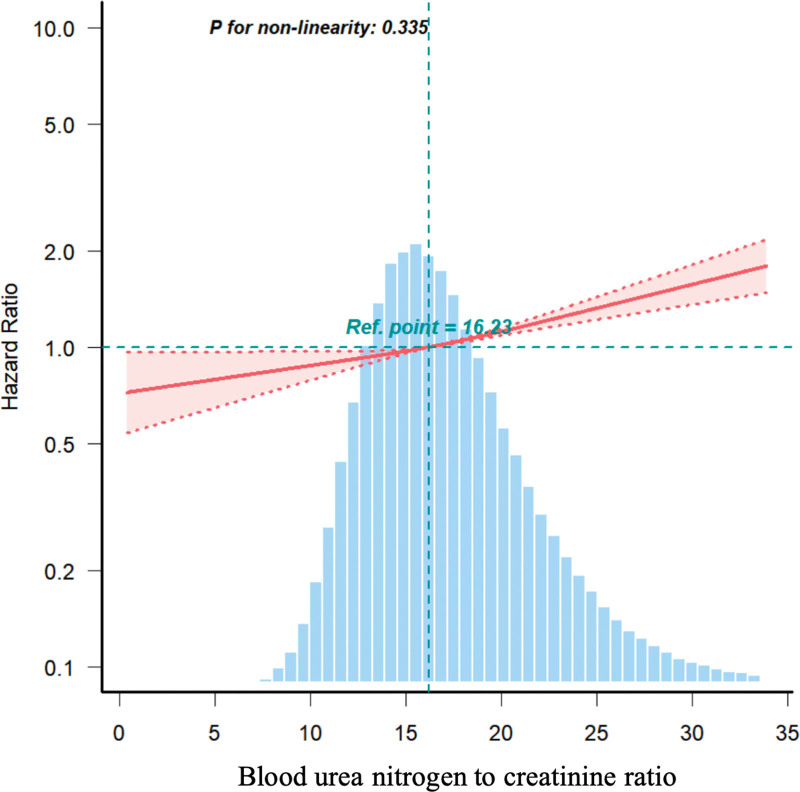
Linear correlation between blood urea nitrogen to creatinine ratio and type 2 diabetes mellitus. The relationship between them was detected based on model IV. The red solid and dashed lines represent the estimates and their corresponding 95% confidence intervals. Ref = reference.

We further explored the correlation between NCR and T2DM by conducting multivariate restricted cubic spline analysis. The results revealed a linear correlation between the NCR and T2DM (Fig. 2). Specifically, there was an increasing trend in T2DM with an increasing NCR.

### 3.4. The results of sensitivity analysis

The results indicated that when NCR was considered as a continuous variable, the effect size remained consistent with the results of previous studies (Table 5).

**Table 5:** Association between NCR and type 2 diabetes mellitus in various sensitivity analyses.

Variables	Model a [HR (95% CI]	*P*-value	Model b [HR (95% CI]	*P*-value	Model c [HR (95% CI]	*P*-value
NCR	1.03 (1.02–1.03)	<0.001	1.03 (1.02–1.03)	<0.001	1.03 (1.02~1.04)	<0.001
NCR ≥ 20						
No	Ref		Ref		Ref	
Yes	1.25 (1.16–1.35)	<0.001	1.28 (1.18~1.38)	<0.001	1.28 (1.19–1.38)	<0.001
NCR (quartile)						
Q1	Ref		Ref		Ref	
Q2	1.07 (0.97–1.18)	0.164	1.06 (0.96–1.17)	<0.001	1.07 (0.97–1.18)	0.178
Q3	1.13 (1.02–1.24)	0.016	1.13 (1.02–1.25)	0.016	1.15 (1.04–1.27)	0.007
Q4	1.36 (1.23–1.5)	<0.001	1.39 (1.25–1.53)	<0.001	1.4 (1.27–1.55)	<0.001
*P*_trend_		<0.001		<0.001		<0.001

Model a was an analysis in individuals without a family history of diabetes (N = 185,453). Age, sex, SBP, BMI, DBP, TG, TC, ALT, AST, smoking status, and drinking status were adjusted for.

Model b was an analysis for never smokers (N = 176,210). Age, sex, SBP, BMI, DBP, TG, ALT, TC, AST, family history of diabetes, and drinking status were adjusted for.

Model c was an analysis for never drinkers (N = 179,924). Age, sex, SBP, BMI, DBP, TG, ALT, TC, AST, family history of diabetes, and smoking status were adjusted for.

ALT = alanine aminotransferase, AST = aspartate aminotransferase, BMI = body mass index, CI = confidence interval, DBP = diastolic blood pressure, HR = hazard ratio, NCR = blood urea nitrogen to creatinine ratio, Ref = reference, SBP = systolic blood pressure, Scr = serum creatinine, TC = total cholesterol, TG = triglyceride.

After adjusting for confounding covariates, we conducted sensitivity analyses on individuals without a family history of diabetes and found a positive connection between NCR and the incidence of T2DM (HR = 1.03, 95% CI: 1.02–1.03, *P* < .001) (Table 5). In addition, our analysis excluding smokers (both current and ever smokers) exhibited a positive association between NCR and the incidence of T2DM (HR = 1.03, 95% CI: 1.02–1.03, *P* < .001). Furthermore, our analysis excluding drinkers (both current and ever drinkers) revealed a consistent positive association between NCR and the incidence of T2DM (HR = 1.03, 95% CI: 1.02–1.04, *P* < .001) (Table 5).

Owing to substantial missing data (approximately 70% for smoking and alcohol status and approximately 50% for AST), we excluded smoking status, alcohol status, and AST from the multivariate model. Despite these exclusions, the results remained consistent with previous findings (HR = 1.03, 95% CI: 1.02–1.04) (Table 4). The findings exhibited high consistency and robustness according to sensitivity analyses.

### 3.5. Subgroup analysis

**Figure 3.**
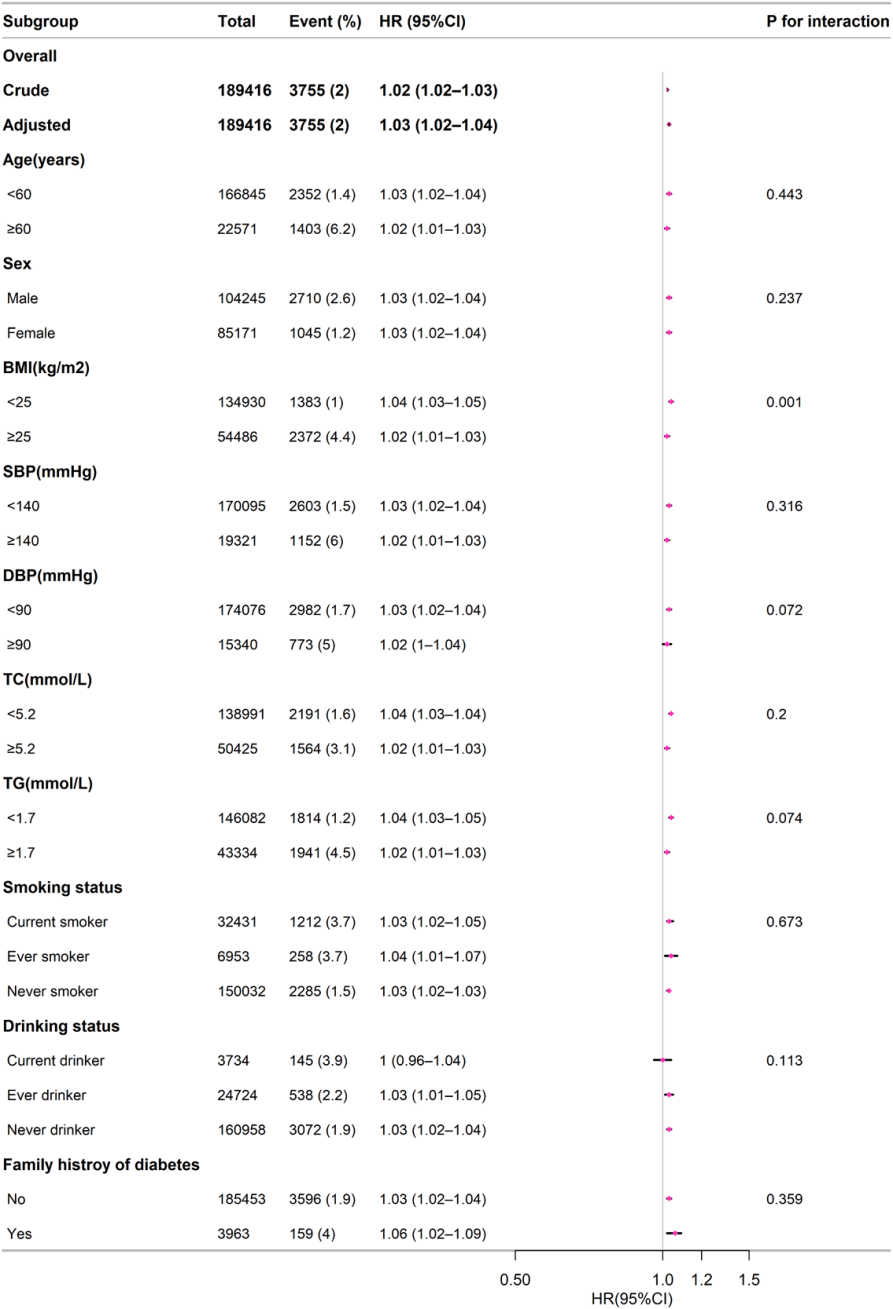
Stratified relationship between blood urea nitrogen to creatinine ratio and type 2 diabetes mellitus. The analysis was based on the model IV. The model did not account for the stratification variable in all cases. BMI = body mass index, CI = confidence interval, DBP = diastolic blood pressure, HR = hazard ratio, SBP = systolic blood pressure, TC = total cholesterol, TG = triglyceride.

A subgroup analysis was performed (Fig. 3). Our analysis demonstrated a consistent positive association between NCR and the incidence of T2DM across all subgroup variables. Considering the multiple testing, a *P*-value < 0.05 for BMI interaction may not be statistically significant.

## 4. Discussion

We found a direct connection between NCR and an increased incidence of T2DM in our retrospective study. Our multivariate models, which considered various confounding factors, revealed that the incidence of T2DM increased by 3% for every unit increase in the NCR. Furthermore, the higher NCR groups (≥20) exhibited a significantly higher incidence of T2DM (HR = 1.28, 95% CI: 1.18–1.38, *P* < .001) compared with the lowest NCR group (<20) after adjusting for confounding factors.

The kidneys play a vital role in the maintenance of glucose homeostasis.^[31,32]^ Moreover, insulin and insulin receptors are involved in renal function and the regulation of glucose homeostasis.^[6–9]^ Insulin resistance is common in CKD.^[6,33]^ At the same time, research showed that patients with CKD and T2DM had common risk factors, and CKD can increase the incidence of T2DM.^[13]^ A cohort study showed that CKD was a substantial and independent indicator of T2DM (HR = 1.204, 95% CI: 1.11–1.31).^[34]^

BUN, Scr, and NCR are commonly used to assess renal dysfunction caused by various factors. Both BUN and Scr undergo glomerular filtration; however, Scr is not reabsorbed by renal tubules. Approximately 40% to 60% of BUN is estimated to be reabsorbed by the tubules.^[35]^ Several recent studies have extensively investigated the relationship between BUN and Scr levels and T2DM. In a study involving 38,578 Chinese individuals who underwent health examinations, BUN was positively correlation with the incidence of T2DM.^[36]^ Similarly, a comprehensive study of United States veterans observed that for every 10 mg/dL rise in BUN concentration, the incidence of T2DM increased by 9% (8%–10%).^[37]^ A cohort study by the Yuport Health Checkup Center found a correlation between low Scr and a higher incidence of T2DM in both males and females.^[38]^ Nevertheless, favorable outcomes from several studies indicate that BUN and Scr levels could be valuable in evaluating the incidence of T2DM.

Nevertheless, BUN is not an exclusive indicator of renal insufficiency and can be influenced by diverse factors such as neurohormonal activation, protein intake, and catabolic processes.^[39]^ Similarly, extrarenal factors, including sex, age, nutrition, and ethnicity, can influence Scr levels.^[15]^ Hence, there may be limitations to making predictions based solely on BUN or Cr levels. The NCR is a valuable parameter for mitigating the factors affecting accuracy. It is considered more reliable and precise than measuring Scr or BUN levels separately.^[16]^ In addition, NCR has been used in clinical practice to differentiate between prerenal and intrinsic renal injury.^[16,17]^ When NCR ≥ 20 is commonly associated with prerenal diseases, which are conditions that reduce blood perfusion to the kidneys and lead to renal dysfunction.^[18,19]^ These prerenal diseases include heart and gastrointestinal diseases.^[40]^ The results of this study showed that the proportion of males was higher in the NCR ≤ 19.638 population, which may be related to the fact that testosterone destroys the kidneys^[41]^ and that some males adopt unprotected anal intercourse^[42]^ to increase the risk of intrinsic or postrenal kidney injury. The proportion of females was higher in the NCR > 19.638 population, which may be related to the higher incidence of cardiac surgery-related kidney injury^[43]^ and nephropathy during pregnancy in females.^[44]^ The above disorders are caused by prerenal kidney injury due to inadequate blood perfusion to the kidneys. Therefore, the reason for sex differences in the NCR population may be related to the etiology of renal injury, but the specific mechanisms need to be further explored.

Our study found that groups with a higher NCR (≥20) showed a higher incidence of T2DM (HR = 1.28, 95% CI: 1.18–1.38, *P* < .001) compared with the lowest NCR group (<20) after adjusting for confounders. These results suggest that there may be a correlation between higher NCR levels and the incidence of T2DM. Our study provided new findings on the relationship between NCR and T2DM. This finding was of great value and provided a valuable resource for the prevention of T2DM in patients with different renal dysfunctions.

We postulated possible mechanisms to explain the correlation between NCR and T2DM. Many studies have revealed that renal dysfunction can damage the tissue sensitivity to insulin. Urea, oxidative stress, activation of the renin–angiotensin–aldosterone system, inflammation, and metabolic acidosis after renal injury may be the underlying mechanisms leading to insulin resistance. ^[34,45,46]^ D’Apolito et al^[47]^ reported a notable correlation between elevated urea and oxidative stress inducement, resulting in insulin resistance and β-cell dysfunction. Renal hypoperfusion can activate the renin–angiotensin–aldosterone system. Many studies have established a connection between elevated aldosterone levels and insulin resistance. Activation of aldosterone-induced mineralocorticoids can negatively impact insulin secretion and sensitivity, which are crucial factors in the progression of T2DM.^[48,49]^ Joshua et al^[50]^ suggested that an increase in log-aldosterone levels was connected with a 44% higher occurrence of T2DM (*P* < .01). Hosoya et al^[51]^ found that in 200 patients with CKD, serum aldosterone levels were associated with the homeostasis model assessment of insulin resistance levels, and treatment with an aldosterone antagonist (spironolactone) can improve the patient’s systemic insulin resistance. Therefore, changes in the NCR may reflect the influence of some kidney diseases on T2DM.

### 4.1. Strengths and limitations

This study has several advantages. The results of this study indicated a positive correlation between NCR and T2DM, which contributed significantly to the existing knowledge on risk factors associated with T2DM. The follow-up period was extended to 6 years, enhancing the credibility of the findings. Sensitivity and subgroup analyses were done to estimate the reliability of the results.

This study has some disadvantages: The study defined T2DM based on FPG and self-report, which may have underestimated the prevalence of T2DM. However, previous research has established a direct correlation between glycosylated hemoglobin and FPG^[52]^ and the substantial sample size helped mitigate this limitation. Our study exclusively focused on the population residing in the urban areas of Southern China, therefore, our findings can be generalized to the Southern Chinese population. However, additional studies are required to determine the northern and non-Chinese populations. In the present study, we only collected baseline measurements of NCR and other parameters. In future research, additional influencing factors, such as changes in NCR during patient follow-up, should be considered.

## 5. Conclusions

Based on a substantial sample population in China, this retrospective cohort study found a positive and independent association between NCR and the likelihood of developing T2DM even after accounting for other confounding factors. Consequently, NCR could serve as a valuable diagnostic tool for detecting T2DM and could offer valuable insights for preventing the incidence of T2DM in patients with varying degrees of renal dysfunction.

## Acknowledgments

We express our gratitude to Dr Qilin Yang from the Department of Critical Care at the Second Affiliated Hospital of Guangzhou Medical University for their valuable contributions in providing statistical support, offering guidance on study design, and providing insightful comments.

## Author contributions

**Conceptualization:** Xiuping Yin.

**Data curation:** Xiuping Yin, Jianjun Jiang.

**Formal analysis:** Xiuping Yin, Fengxing Zhong.

**Methodology:** Xiuping Yin, Yiguo Wang.

**Writing—original draft:** Xiuping Yin.

**Supervision:** Yiguo Wang.

**Writing—review and editing:** Qiming Zhang.
